# The leader of the capsid protein from *Feline calicivirus* must be palmitoylated and form oligomers through disulfide bonds for efficient viral replication

**DOI:** 10.1128/jvi.01270-25

**Published:** 2025-08-19

**Authors:** Yoatzin Peñaflor-Téllez, Jaury Gómez de la Madrid, Erick Ignacio Monge-Celestino, Carolina Pérez-Ibáñez, Randy Seir Llanas Vázquez, Sharon Itzel Sosa Mondragon, Carlos Emilio Miguel-Rodríguez, Ana Lorena Gutiérrez-Escolano

**Affiliations:** 1 Departamento de Infectómica y Patogénesis Molecular, Centro de Investigación y de Estudios Avanzados del Instituto Politécnico Nacional 42576, Mexico City, Mexico; University of Michigan Medical School, Ann Arbor, Michigan, USA

**Keywords:** *Feline calicivirus*, LC protein, viroporin, palmitoylation, LC secretion, PDIA3, oligomers

## Abstract

Feline calicivirus (FCV), a member of the genus *Vesivirus* and a model for studying members of the family *Caliciviridae*, has been used to understand the biology and viral replication of etiological agents of medical and veterinary importance. The leader of the capsid (LC) protein is exclusive to members of the genus *Vesivirus,* and its importance for successful viral replication has been demonstrated; however, its cellular localization, post-translational modifications, and specific functions throughout the viral replication cycle are poorly understood. Using a virus-free system, we determined that the expression of the LC protein from FCV localizes to the mitochondria and induces apoptosis; furthermore, its *in vitro* characterization showed that it exhibits viroporin-like characteristics. Here, using an LC-reactive serum, we analyzed the kinetics, subcellular localization, post-translational modifications, and association of the LC protein with cellular protein disulfide isomerases (PDIs) in FCV-infected cells. We found that the LC protein is present on the membrane of infected cells and in their supernatants, independently of cell lysis, suggesting that it is actively secreted. Moreover, we found that the LC protein is palmitoylated during infection, and that palmitoylation inhibition alters its levels and subcellular localization. Finally, we demonstrate that the LC protein forms oligomers through disulfide bonds mediated by PDI activity. Given that LC protein is mainly localized to the cytoplasm and plasma membrane, its interaction with PDIA3, a disulfide isomerase primarily located in the endoplasmic reticulum but also present in the cytoplasm and plasma membrane and recognized as an important factor in the replication of several viruses such as influenza A, dengue, and Zika virus, may be responsible for LC dimerization. All these results indicate that the LC protein from FCV might have multiple roles during FCV replication.

**IMPORTANCE:**

Feline calicivirus (FCV) is a highly transmissible virus that represents a significant cause of upper respiratory infection in domestic and wild cats worldwide. FCV also serves as one of the most valuable models for studying calicivirus biology, as unlike most members of the family, known to cause diseases in animals and humans, it can be easily grown in cell culture. Since there are no efficacious vaccines or antivirals against most caliciviruses, understanding their molecular biology and the relationship between viral and cellular components is essential for developing strategies for their prevention and control.

## INTRODUCTION

Feline calicivirus (FCV), a member of the genus *Vesivirus* in the family *Caliciviridae*, efficiently replicates in cell culture and has multiple reverse genetic systems. For the last 70 years, it has been one of the most important models for understanding the replication strategies of members of the family *Caliciviridae* in infected cells (1). FCV is an etiological agent of upper respiratory tract pathologies in domestic and wild cats (2). In some cases, FCV infection may lead to a highly lethal systemic disease that can easily spread in shelters or veterinary clinics (3–6). Several FCV vaccines are available, but while they can greatly reduce the severity of infection, they do not provide full protection against FCV. In addition, there is no approved antiviral treatment for FCV (7–9), making it an important veterinary and wildlife concern.

Caliciviruses are non-enveloped RNA viruses with positive-sense, single-stranded genomes that encode six non-structural (NS1–NS7) and two structural (VP1 and VP2) proteins. Although all viral proteins are encoded in the genomic RNA, the structural proteins are translated late in infection from a subgenomic RNA (sgRNA) (10). A notable difference between vesiviruses, such as FCV, and other members of the family *Caliciviridae* is the presence of a unique protein named the leader of the capsid (LC), which is translated from the sgRNA as an LC-VP1 precursor. This precursor is further processed by the viral protease-polymerase NS6/7 to generate both the mature LC and the major capsid protein VP1 (11). Although the LC does not form part of the mature FCV virions (12), it plays a significant role during infection.

In 2013, Abente et al. described that the LC protein is crucial for establishing the cytopathic effect during FCV infection and overall virus replication (13). The same research group found that FCV LC protein interacts with annexin A2 (ANXA2), a protein that our research group previously identified as an important cellular factor for efficient replication (14). We later determined that its ectopic expression leads to mitochondrial localization and triggers apoptosis through the intrinsic pathway (15). In a more comprehensive characterization of the LC protein, we found that it is intrinsically toxic and forms homo-oligomers dependent on disulfide bonds (16). Moreover, we recently reported that recombinant LC protein can permeate the plasma membrane of non-infected cells during exogenous interaction (17). These findings led us to suggest that the LC protein from FCV is a viroporin; however, more information regarding its expression, localization, and interaction with cellular components is needed to fully understand its role(s) in the FCV replication cycle.

Here, we describe the expression kinetics of the LC protein during FCV infection. Our findings indicate that its subcellular localization drastically differs from the mitochondrial localization of the LC protein expressed in a virus-free system, as previously reported (15). The LC protein was found in the cytoplasm, on the inner and possibly the outer surfaces of the plasma membrane and in the extracellular medium, suggesting that it is secreted during viral infection. In addition to its subcellular localization, palmitoylation, a post-translational modification of the LC protein, was predicted and further confirmed *in vitro*, with potential implications for its abundance and localization. Given that the LC protein forms disulfide bond-dependent dimers and is mainly localized to the cytoplasm and plasma membrane, we hypothesize that it may interact with a disulfide isomerase present in these compartments, such as protein disulfide isomerase A3 (PDIA3). PDIA3 is a key disulfide isomerase involved in the replication of several RNA viruses (18–22) and, although primarily localized in the endoplasmic reticulum, it has also been detected in other cellular compartments including the cytoplasm, nucleus, and plasma membrane (23). We found that inhibition of PDIs resulted in changes in both the subcellular localization and levels of the LC protein in FCV-infected cells. These results help define some of the characteristics of the LC protein, indicating that it might play multiple roles in FCV-infected cells, consistent with its previously stated importance for successful viral replication cycle.

## RESULTS

### LC protein expression levels and subcellular localization during FCV infection

**Fig 1:**
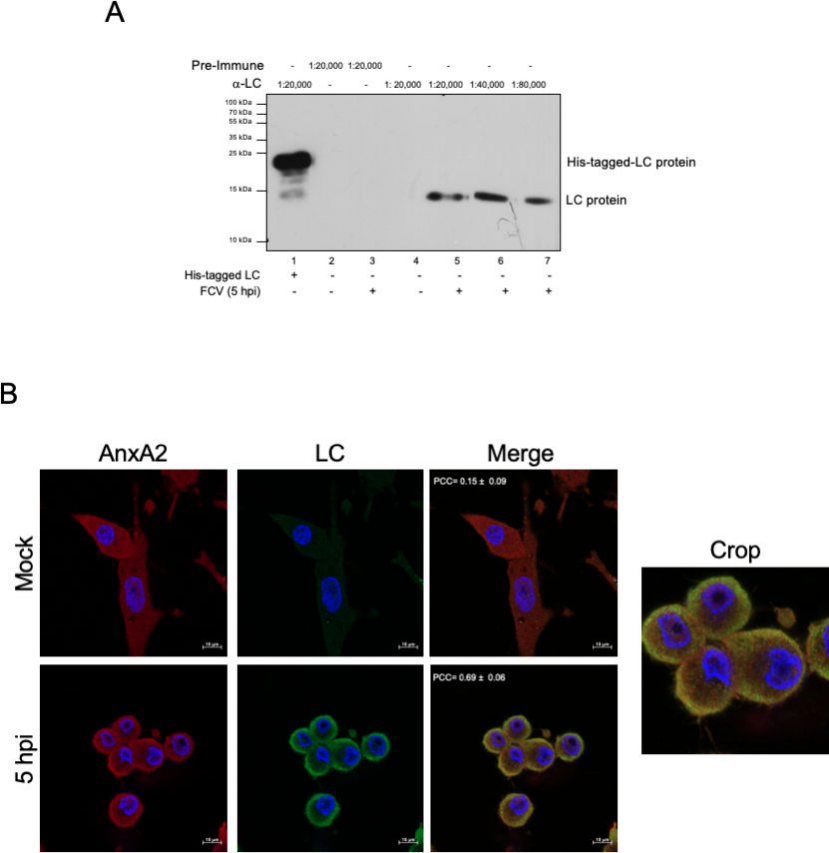
Specificity of the serum against the LC protein from FCV. (A) His-tagged LC recombinant protein (line 1) and total protein extracts from mock-infected (lines 2 and 4) and infected cells at a multiplicity of infection (MOI) of 5 for 5 h (lines 3, 5–7) were obtained, and LC protein was detected by western blotting. Preimmune and anti-LC sera were used at 1:20,000, 1:40,000, and 1:80,000 as indicated. The positions corresponding to the migration of His-tagged LC and LC proteins are indicated. (B) Mock-infected or FCV-infected cells at an MOI of 5 for 5 h were immunostained with an anti-LC serum (green) or with anti-annexin A1 (red). DAPI was used for nuclear (blue) staining. The cells were examined in a Zeiss LSM 700 confocal microscope. Images correspond to a z-stack of 15 slices and represent at least three independent experiments. Merged and cropped images are indicated. Pearson’s correlation coefficients, indicated in the images, were calculated for 15 individual cells from two independent experiments. Data are presented as mean ± SD using Zeiss software (https://www.zeiss.com.mx/corporate/home.html).

It has been previously established that the LC protein from FCV is translated late during infection as an LC-VP1 precursor protein processed by the viral protease-polymerase NS6/7 (11). To determine the time post-infection at which the LC protein was first detected and to elucidate its expression levels during the FCV replicative cycle in CrFK cells, an antibody against LC was generated from a recombinant protein and tested by western blotting to identify LC in extracts from infected cells for 5 h post-infection (hpi) (Fig. 1A). The His-tagged LC recombinant protein was also included as a positive control. The anti-LC serum specifically recognized a single band of approximately 14 kDa in extracts from infected cells, corresponding to the known molecular weight of LC (Fig. 1A, lines 5–7), as well as the 17 kDa His-tagged LC recombinant protein (Fig. 1A, line 1). No reactivity was observed in mock-infected cells or when using the preimmune serum on extracts from either mock-infected or infected cells (Fig. 1A, lines 1, 2, and 4 respectively).

Once the specificity of the generated anti-LC serum was evaluated by western blotting, its ability to recognize the LC protein in infected cells was further validated by immunofluorescence assays. The localization of LC protein was analyzed in FCV-infected cells alongside its known interacting partner: the cellular protein annexin A2 (13). At 5 hpi, annexin A2 (Fig. 1B) colocalized with the LC signal detected by the anti-LC serum, with a Pearson’s coefficient colocalization value of 0.69 ± 0.06, further supporting the antibody’s specificity.

**Fig 2:**
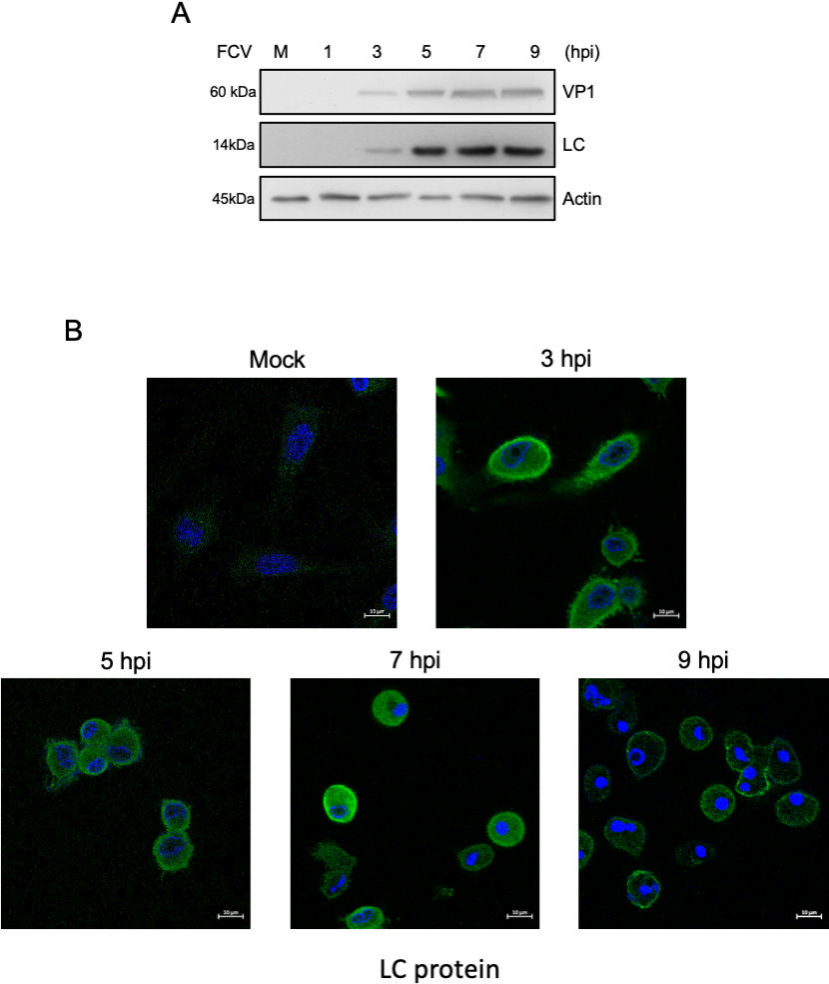
LC protein subcellular localization and expression through the FCV replicative cycle. (A) Total protein extracts from mock-infected or FCV-infected cells at an MOI of 5 for 1, 3, 5, 7, and 9 h were obtained, and levels of LC and VP1 proteins were determined by western blotting. Actin was used as a loading control. (B) Mock-infected or FCV-infected cells at an MOI of 5 for 3, 5, 7, and 9 h were immunostained with the anti-LC serum (green). DAPI was used for nuclear (blue) staining. The cells were examined in a Zeiss LSM 700 confocal microscope. Images correspond to a z-stack of 15 slices and represent at least three independent experiments.

Subsequently, the anti-LC serum was used to assess the expression kinetics and relative expression levels of the LC protein in extracts from FCV-infected cells at a multiplicity of infection (MOI) of 5, harvested at 1, 3, 5, 7, and 9 hpi by western blotting (Fig. 2A). We found that the LC protein was first detected at 3 hpi and up to 9 hpi, reaching its peak expression at 5 hpi and following a similar expression pattern as the VP1 protein (Fig. 2A). To analyze its subcellular distribution during infection, CrFK cells were infected with FCV at an MOI of 5, and the LC protein subcellular distribution was determined at different times post-infection by confocal microscopy (Fig. 2B). At 3 hpi, the LC protein was observed in both the cytoplasm and the periphery of the infected cells, which showed the first signs of cell rounding indicative of a cytopathic effect (Fig. 2B). A similar subcellular localization was observed at 5 and 7 hpi (Fig. 2B), but the signal intensity appeared greater than that observed at 3 hpi, correlating with the highest levels of the protein detected by western blotting (Fig. 2A). At 7 hpi, the LC protein was more evenly distributed throughout the cytoplasm, coinciding with established cytopathic effects and apoptosis (marked by pyknotic nuclei in infected cells) (Fig. 2B). At 9 hpi, the LC protein signal was reduced, correlating with its detection by western blotting (Fig. 2B), and was mainly observed in the cell periphery in clusters that appeared to be located on the cellular membrane (Fig. 2B).

**Fig 3:**
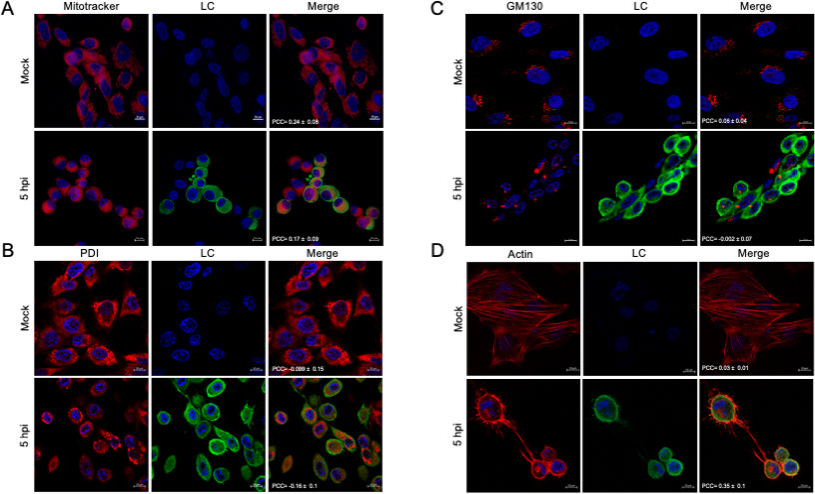
The LC protein is located in the cytoplasm and the cell periphery of FCV-infected cells. Mock-infected or FCV-infected cells at an MOI of 5 for 5 h were immunostained with an anti-LC serum (green) and (A) MitoTracker (red), (B) an anti-PDI (red), (C) an anti-GM130 (red), and (D) an anti-actin (red) antibody. DAPI was used for nuclear (blue) staining. The cells were examined in a Zeiss LSM 700 confocal microscope. Images correspond to a z-stack of 15 slices and represent at least three independent experiments. Merged images are indicated. Pearson’s correlation coefficients, indicated in the images, were calculated for 18 individual cells from two independent experiments. Data are presented as mean ± SD using Zeiss software (https://www.zeiss.com.mx/corporate/home.html).

To further determine the specific subcellular localization of the LC protein in FCV-infected cells, we used subcellular markers of distinct compartments or organelles and analyzed colocalization with the LC protein by confocal microscopy at 5 hpi. To examine the possible localization of the LC protein in the mitochondria during infection, CrFK cells were infected at an MOI of 5, and its colocalization with the mitochondria was determined using MitoTracker staining and analyzed by confocal microscopy (Fig. 3A). A discrete colocalization between the LC protein and MitoTracker-stained mitochondria was detected at 5 hpi with a Pearson’s coefficient colocalization value of 0.17 ± 0.09 (Fig. 3A), in contrast to the pronounced colocalization of the exogenously expressed LC protein in CrFK cells transfected with the LC-pAm-Cyan, but not with the pAM-Cyan plasmid alone, as previously reported by our workgroup (15). The observed difference in LC localization between its expression during infection and in a virus-free system has also been reported for other viral proteins (24–26).

To determine whether the LC protein localizes to the endoplasmic reticulum (ER) or the Golgi apparatus during infection, its colocalization with PDI, an ER-resident luminal protein and marker of FCV replication complexes (RCs) (27), and GM130, a resident of the Golgi apparatus, was analyzed by confocal microscopy (Fig. 3B and C). No colocalization was observed with either marker as the Pearson’s coefficient colocalization values were −0.16 ± 0.1 and −0.002 ± 0.07, respectively, suggesting that the LC protein is not significantly associated with these organelles.

Since the LC protein is also observed in the periphery of infected cells (Fig. 2B), it is possible that it is associated with cortical actin, an important component of the cell cytoskeleton and a key regulator of multiple cellular signaling pathways. Colocalization analysis revealed a weak association between the LC protein and actin at the periphery of the infected cells, with a Pearson’s coefficient colocalization value of 0.35 ± 0.1 (Fig. 3D). These results demonstrate that the LC protein expression increases steadily from 3 hpi, reaching a maximum at 5 hpi. LC protein localizes near the RC and at the cell periphery, most probably in the cytoplasm and potentially in association with cortical actin, but not with mitochondria, the ER, or the Golgi apparatus.

### LC of FCV is located on the inner and possibly in the outer faces of the plasma membrane

**Fig 4:**
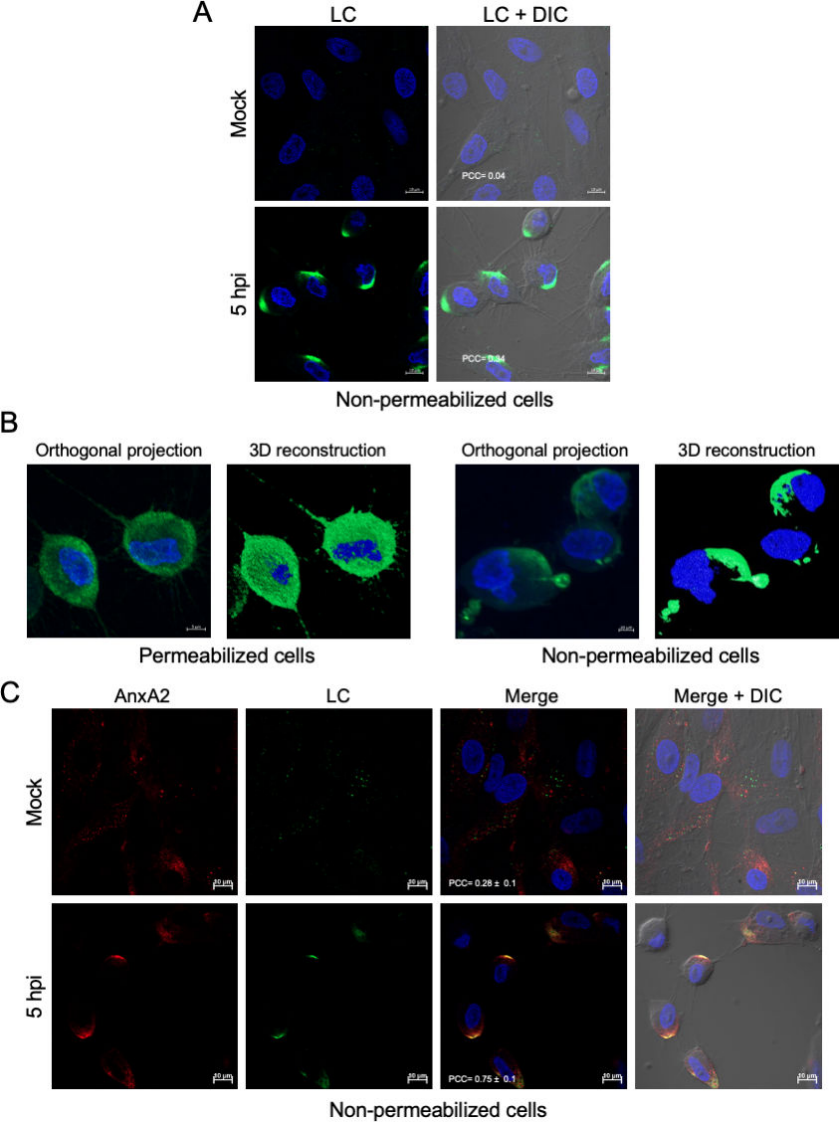
The LC protein is probably located on the outer face of the plasma membrane during FCV replication. (A) Non-permeabilized, mock-infected, or FCV-infected cells at an MOI of 5 for 5 h were immunostained with an anti-LC serum (green). (B) Orthogonal projections and 3D reconstructions from confocal microscopy assays of permeabilized and non-permeabilized FCV-infected cells. (C) Non-permeabilized, mock-infected, or FCV-infected cells at an MOI of 5 for 5 h were immunostained with anti-LC serum (green) and anti-annexin A2 antibody. DAPI was used for nuclear (blue) staining. The cells were examined in a Zeiss LSM 700 confocal microscope. Images correspond to a z-stack of 15 slices and represent at least three independent experiments. Merged images are indicated. Pearson’s correlation coefficients, indicated in the images, were calculated for 16 individual cells from two independent experiments. Data are presented as mean ± SD using Zeiss software (https://www.zeiss.com.mx/corporate/home.html).

Since the LC protein was detected in the cell periphery during different times of infection and appeared to colocalize with cortical actin, it is likely that this viral protein was also located on the outer face of the plasma membrane. To evaluate this possibility, its localization was assessed in non-permeabilized, FCV-infected cells by confocal microscopy at 5 hpi (Fig. 4A). In non-permeabilized cells, the LC protein exhibited a focalized, capping-like distribution pattern, in contrast to its homogenous distribution along the inner face of the plasma membrane observed in permeabilized cells (Fig. 2C, 4A and B).

Given that annexin A2 is also located on the extracellular face of the plasma membrane (28) and has been shown to associate with the LC protein, the colocalization of both proteins was examined in non-permeabilized cells at 5 hpi by confocal microscopy (Fig. 4C). Both proteins were observed to colocalize within the same focalized regions, as the Pearson’s coefficient colocalization value was 0.75 ± 0.1, suggesting the presence of the LC protein on the outer face of the plasma membrane. These findings strongly suggest that, in addition to its localization on the inner face of the plasma membrane, the LC protein is also specifically present on its outer surface.

### LC protein from FCV is palmitoylated in FCV-infected cells

Once the localization of the LC protein at the plasma membrane was established, we sought to investigate how it reaches this localization, considering that *in silico* analyses previously performed by our workgroup had suggested the presence of a transmembrane domain (TMD) within the C-terminal region of the FCV LC protein (17). A common determinant for proteins to localize to specific subcellular membranous compartments is through lipidation, which provides a hydrophobic anchor for embedding in its target membrane. Among lipidations, palmitoylation is a reversible post-translational modification carried out through the activity of palmitoyl acyltransferases or zDHHC enzyme family members, directing the specific subcellular localization of target proteins. There are multiple reports of viral proteins that are palmitoylated by the zDHHC palmitoyl transferases of their hosts, impacting their localization and/or stability (reviewed in references 29, 30).

**Fig 5:**
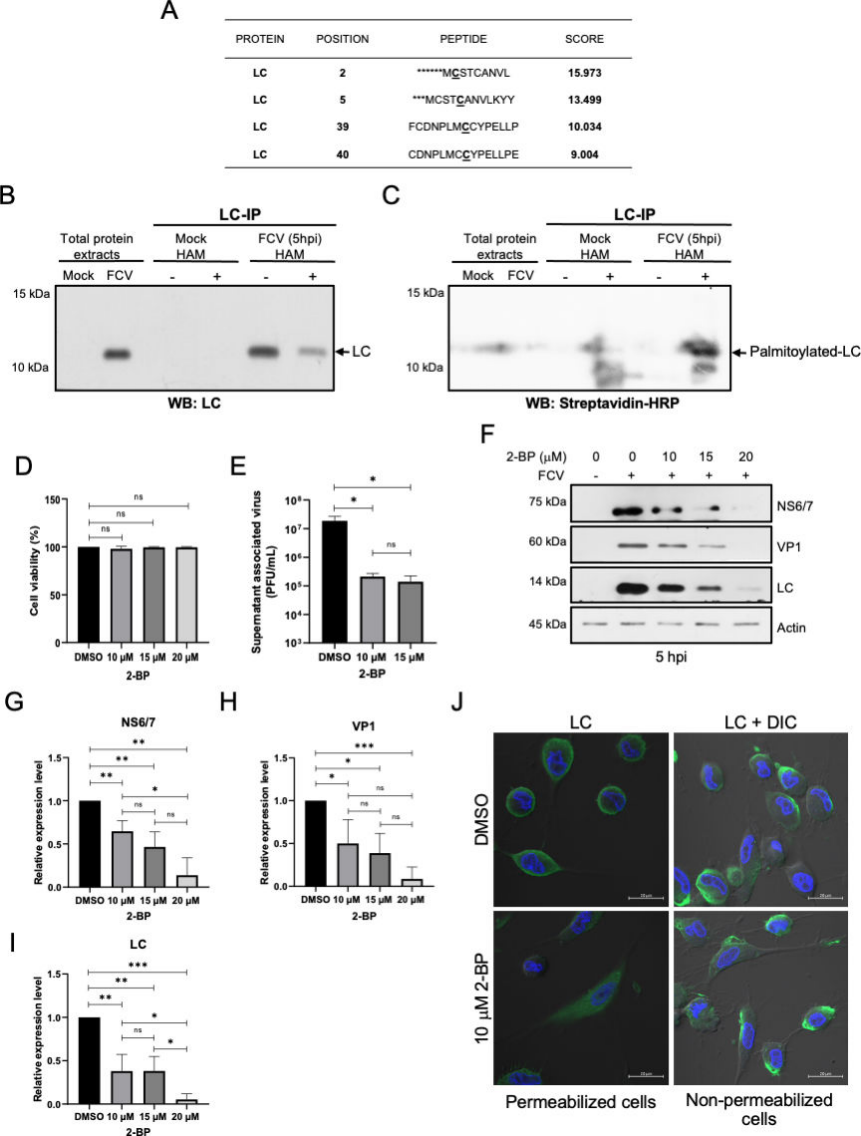
The LC protein form FCV is palmitoylated during infection. (A) Putative cysteine residues susceptible to palmitoylation of the LC protein from FCV were identified using the GPS Palm 4.0 software. Total protein extracts from mock-infected or FCV-infected cells at an MOI of 5 for 5 h were obtained and subjected to immunoprecipitation with an anti-LC serum and subjected to ABE; the LC protein was detected by western blotting using (B) anti-LC antibody or (C) streptavidin-HRP. Hydroxylamine non-treated samples were used as an internal control. (D) CrFK cells were treated with 10, 15, and 20 µM of 2-BP, and cell viability was assessed by an MTT assay. (E) CrFK cells treated with 10 or 15 µM of 2-bromopalmitate were infected with FCV at an MOI of 5 for 5 h, and the supernatant-associated virus yield was quantified by plaque assay. Standard deviations were obtained from three independent experiments. Values of *P* < 0.05 (*) calculated by *t*-test using GraphPad Prism 8.0 software are indicated. (F) Total protein extracts from cells treated with 10, 15, and 20 µM of 2-bromopalmitate, and mock-infected or infected with FCV at an MOI of 5 for 5 h, were obtained, and levels of NS6/7, VP1, and LC proteins were determined by western blotting. Actin was used as a loading control. (G) NS6/7, (H) VP1, and (I) LC band intensities from scanned images were quantified using ImageJ software and shown as relative expression. Standard deviations were obtained from three independent experiments. Values of *P* < 0.05 (*), *P* < 0.001 (**), and *P* < 0.0005 (***) calculated by *t*-test using GraphPad Prism 8.0 software are indicated. (J) CrFK cells treated with DMSO or 10 µM 2-BP were infected with FCV at an MOI of 5 for 5 h, permeabilized or non-permeabilized, and immunostained with an anti-LC serum. The cells were examined in a Zeiss LSM 700 confocal microscope. Images correspond to a z-stack of 15 slices and represent at least three independent experiments.

To determine if the LC protein undergoes palmitoylation, we looked for putative palmitoylation signals in its primary structure using the palmitoylation prediction server GPS Palm v.4.0 (31) and found that the LC protein contains four cysteine residues at positions 2, 5, 39, and 40 that could be S-palmitoylated. (Fig. 5A).

To confirm that the LC protein was palmitoylated during FCV viral replication, it was immunoprecipitated from infected cell protein extracts and subjected to an acyl-biotin exchange (ABE) assay (Fig. 5B and C). The specific immunoprecipitation of the LC protein from infected cells at 5 h was performed using an anti-LC serum, either in the presence or absence of hydroxylamine (HAM), and detected by western blotting using the anti-LC serum (Fig. 5B). A positive signal for the LC protein was found in the total protein extracts from infected cells and in the immunoprecipitated fraction from the extracts with or without HAM (Fig. 5B). A reduction in the intensity of the LC band in the presence of HAM is due to partial degradation caused by HAM treatment, as previously reported (32). To determine if the LC protein was palmitoylated, a parallel western blotting using streptavidin-horseradish peroxidase (HRP) was performed (Fig. 5C). The immunoprecipitated LC protein from infected cell extracts treated with HAM, the only condition that allowed the ABE to occur, but not the immunoprecipitated LC protein from infected cell extracts not treated with HAM, was detected by streptavidin-HRP (Fig. 5C), indicating that this protein is palmitoylated during FCV replicative cycle.

To determine if the LC protein palmitoylation is required for efficient viral replication and could be related to its subcellular localization, CrFK cells were treated with different concentrations of the zDHHC palmitoyl transferases inhibitor 2-bromopalmitate (2-BP) and infected with FCV at an MOI of 5 for 5 h. The supernatant viral titers, early and late viral protein production, and subcellular localization of LC protein were analyzed by plaque assays, western blotting, and immunofluorescence (Fig. 5E through J). Viability of CrFK cells treated with 10, 15, and 20 µM of 2-BP for 5 h was determined by 3-(4,5-dimethylthiazol-2-yl)-2,5-diphenyltetrazolium bromide (MTT) assays (Fig. 5D), and no significant toxicity was observed at any of the concentrations used compared to the inhibitor vehicle dimethyl sulfoxide (DMSO). Next, we analyzed the viral progeny present in the supernatant of drug-treated, FCV-infected CrFK cells. A 2-log reduction in viral titers from infected cells treated with 10 and 15 mM of 2-BP was found in comparison with the control vehicle-treated cells at 5 hpi (Fig. 5E). To determine in which step of the viral cycle palmitoylation of the LC protein was required, the synthesis of early and late viral proteins in the presence or absence of 2-BP was analyzed by western blotting. A significant reduction of the early NS6/7 and late LC and VP1 viral proteins in cells infected for 5 h and treated with 10 and 15 µM of 2-BP was found, compared to the infected DMSO-treated cells (Fig. 5F through I). This suggests that the LC protein palmitoylation is required from the early stages for efficient FCV replication.

Once we demonstrated that palmitoylation inhibition affected viral protein levels and virus yield, we wanted to determine if this post-translational modification plays a role in the localization of the LC protein to the cell periphery and the external face of the plasma membrane during infection. Therefore, infected CrFK cells treated with 10 µM 2-BP, a concentration at which a reduction but not a complete depletion of the LC protein levels was observed (Fig. 5J), exhibited a markedly more heterogeneous phenotype in which the typical cell-rounding morphology was absent, and the LC protein was detected in the cytoplasm and, to a lesser extent, in the cell periphery (Fig. 5J). In contrast, no differences regarding the LC protein localization were observed between treated and non-treated, non-permeabilized cells (Fig. 5J). These results suggest that the palmitoylation of the LC protein and cellular proteins influences, to some extent, the intracellular localization of the LC protein but is not required for its localization on the cell surface. We cannot rule out the possibility that the changes in the intracellular localization of LC in the presence of 10 µM 2-BP could be due to the reduced protein abundance that resembles early stages of infection. All these results, taken together, indicate that the LC protein is palmitoylated in FCV-infected cells and that palmitoylation influences the intracellular localization of the LC protein, but not its traffic to the outer face of the plasma membrane.

### LC protein is secreted to the extracellular medium

**Fig 6:**
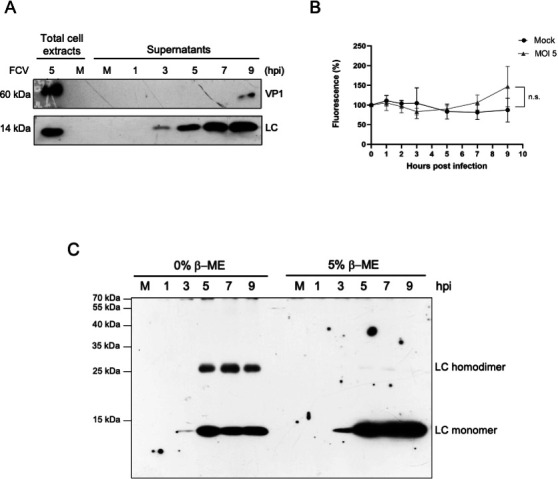
The LC protein can homodimerize and be secreted from FCV-infected cells. (A) CrFK cells were mock-infected or FCV-infected at an MOI of 5 for 1, 3, 5, 7, and 9 h, and the presence of the LC and VP1 proteins in the supernatants was determined by western blotting. Total protein extracts from mock-infected and FCV-infected cells at an MOI of 5 for 5 h were included as controls. (B) Mock-infected and FCV-infected cells at an MOI of 5 for 5 h were incubated with Sytox Green for 10 min, and the total fluorescence was determined using the microplate fluorometer Fluoroskan Ascent FL. Standard deviations were obtained from three independent experiments. (C) Total extracts from mock-infected and FCV-infected cells at 1, 3, 5, 7, and 9 h in the absence or presence of 5% β-ME were obtained, and the levels of the LC protein monomer and dimer were determined by western blotting. LC protein monomers and dimers are indicated.

Given the localization of the LC protein at the cell periphery and the outer face of the plasma membrane in FCV-infected cells and its interaction with annexin A2 (13), a protein involved in secretion, we investigated the possibility of the LC protein secretion during infection. The LC protein was detected in the supernatants of infected cells from 3 hpi, the same time it was first detected by western blotting (Fig. 6A). This corresponds to the time at which the LC protein was first detected in a whole cell protein extract from infected cells (Fig. 2A). Moreover, the LC protein was also detected in increasing concentrations in the supernatants collected at 5, 7, and 9 hpi. To rule out the possibility that the presence of the LC protein in the supernatants was due to cell membrane disruption or cell lysis, a Sytox Green entry assay was performed to evaluate cell permeation hourly up to 9 hpi using the microplate fluorometer Fluoroskan Ascent FL (Fig. 6B). No statistically significant changes in cell membrane permeation were observed at any of the analyzed times, including those at which the LC protein is located outside the infected cell and in the supernatant (Fig. 6B). Furthermore, no monomeric VP1 was detected in the supernatants of infected cells up to 7 hpi. These results strongly suggest that the LC protein is actively secreted during the FCV replication cycle and may act as an extracellular viroporin.

### LC from FCV forms homo-oligomers through disulfide bond formation

Viroporins often contain disulfide bonds that contribute to their structure, stability, or function (33–36). We have previously reported that purified His-tagged LC protein homo-oligomerizes through disulfide bond formation; however, the oligomeric state of the LC protein in FCV-infected cells was still undetermined. The oligomerization state of the LC protein during FCV replication cycle, and its formation dependence on disulfide bonds, was determined at 1, 3, 5, 7, and 9 hpi under non-reducing and reducing conditions using 5% β-mercaptoethanol (β-ME) as a reducing agent (Fig. 6C). In the non-reduced protein extracts, both a 14 kDa band corresponding to the monomeric LC and a band of 28 kDa corresponding to the homo-dimeric LC protein were detected at 5, 7, and 9 hpi, while in 5% β-ME-treated protein extracts collected at the same times post-infection, only an enriched band of 14 kDa corresponding to the monomeric form was detected, indicating that under reducing conditions, the dimeric form was dissociated into monomeric forms (Fig. 6C). These results indicate that the LC protein forms homodimers through disulfide bonds in FCV-infected cells.

### The LC protein from FCV interacts with the protein disulfide isomerase A3

**Fig 7:**
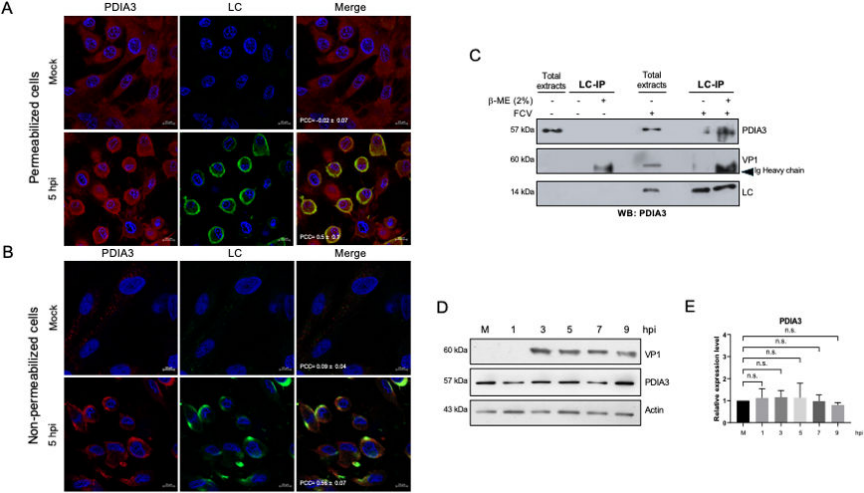
LC interacts with PDIA3 in FCV-infected cells. (A) Permeabilized and (B) non-permeabilized mock-infected and FCV-infected cells at an MOI of 5 for 5 h were immunostained with an anti-LC serum (green) and anti-PDIA3 (red) antibodies. DAPI was used for nuclear (blue) staining. The cells were examined in a Zeiss LSM 700 confocal microscope. Images correspond to a z-stack of 15 slices and represent at least three independent experiments. Merged images are indicated. Pearson’s correlation coefficients, indicated in the images, were calculated for 25 individual cells from two independent experiments. Data are presented as mean ± SD using Zeiss software (https://www.zeiss.com.mx/corporate/home.html). (C) Total protein extracts from mock-infected or FCV-infected cells at an MOI of 5 for 5 h were obtained and subjected to immunoprecipitation with an anti-LC serum. LC, VP1, and PDA3 proteins were detected by western blotting. Elutions were performed in the absence or presence of 2% β-ME + 5 min heating at 95°C. Total protein extracts from mock-infected and FCV-infected cells at 5 h were included as controls. The positions corresponding to the migration of the heavy chain from the mouse IgG are indicated. (D) Total protein extracts from mock-infected or FCV-infected cells at an MOI of 5 for 1, 3, 5, 7, and 9 h were obtained, and levels of PDIA3 were determined by western blotting. VP1 indicates a virus infection. Actin was used as a loading control. (E) PDIA3 band intensities from scanned images were quantified using ImageJ software and shown as relative expression. Standard deviations were obtained from three independent experiments.

We demonstrated that the LC protein forms disulfide bond-dependent homodimers in FCV-infected cells. Since the LC protein does not appear to localize to the ER or Golgi apparatus but is instead found in the cytoplasm and the plasma membrane of infected cells and is also secreted, we hypothesized that PDIA3—an abundant protein with a significant fraction residing in the cytosol, also present on both sides of the plasma membrane, and known to play multiple roles in the replication cycle of various viruses (19–23)—may be involved in LC protein function. Colocalization between the LC protein and PDIA3 was observed mainly in the plasma membrane in permeabilized FCV-infected cells with a Pearson’s coefficient colocalization value of 0.5 ± 0.1 (Fig. 7A), demonstrating proximity between these two proteins at the cell periphery. Notably, higher levels of PDIA3 were observed on the plasma membrane of the FCV-infected, non-permeabilized cells, and in close contact with LC than in mock-infected, non-permeabilized cells, with a Pearson’s coefficient colocalization value of 0.56 ± 0.07, which suggests that in FCV-infected cells, PDIA3 is in close contact with LC (Fig. 7B). This suggested association between the LC and PDIA3 proteins was corroborated by immunoprecipitating the LC protein and analyzing the obtained immunocomplexes by matrix-assisted laser desorption/ionization time-of-flight tandem mass spectrometry (MALDI-TOF/TOF) (Table 1). Among the proteins identified as associated with the LC protein but absent in the mock control, we found ANXA2, which has previously been reported to interact with the LC protein (13); heat shock protein 90 beta (HSP90B), which has been described as being involved in FCV replication (37); and additional proteins such as Tripartite motif-containing 21 (TRIM21) and core histone H2B (H2B), whose roles in FCV replication remain unknown. Several actin isoforms, including β-actin, were also detected in mock-infected samples, probably due to their high abundance and adhesive nature (Table 1).

**TABLE 1:** Cellular proteins associated with LC, identified by mass spectrometry in FCV-infected CrFK cells at 5 hpi

NCBI reference sequence	Protein	Protein score	Coverage (%)	Matched peptides
NP_001092.1	ACTB^*a*^	12,128.26	65.12	19
NP_001002857.1	ANXA2	1,043.658	29.2	9
NP_003290.1	HSP90B1	1,674.934	31.77	23
NP_003516.1	H2B	11,295.54	35.2	7
NP_005304.3	PDIA3	438.9395	17.62	11
NP_003132.2	TRIM21	2,808.353	26.01	10
	FCV-capsid	3,673.5990	46.11	22

^*a*^ Proteins associated with LC that were also found in mock-infected cells.

The association between the LC protein and PDI3A was confirmed by immunoprecipitation from cell lysates collected at 5 hpi and analyzed by western blotting under reducing and non-reducing conditions (Fig. 7C). PDI3A was detected in both mock-infected and FCV-infected total protein extracts, while the LC protein and VP1, included as a control, were detected only in infected cells (Fig. 7C). PDIA3 was co-immunoprecipitated with the LC protein, as it is strongly detected in the immunoprecipitation fraction from FCV-infected cells under reducing conditions, indicating its association with the LC protein during FCV infection.

To determine the relative expression of PDIA3 during infection, total protein extracts from mock-infected and FCV-infected cells at 1, 3, 5, 7, and 9 h were obtained, and the levels of PDIA3 were analyzed by western blotting (Fig. 7D and E). Similar PDIA3 levels were observed in both mock-infected and FCV-infected protein extracts at different times, indicating that this protein is not modulated during FCV replication (Fig. 7D and E). Taken together, these results demonstrate that the LC protein from FCV interacts with PDIA3, an important disulfide isomerase protein that participates in the replication cycle of various viruses, and that PDIA3 levels are not modulated during FCV infection.

### PDIA3 protein is involved in the localization of the LC protein in the periphery of FCV-infected cells and in the formation of oligomers

**Fig 8:**
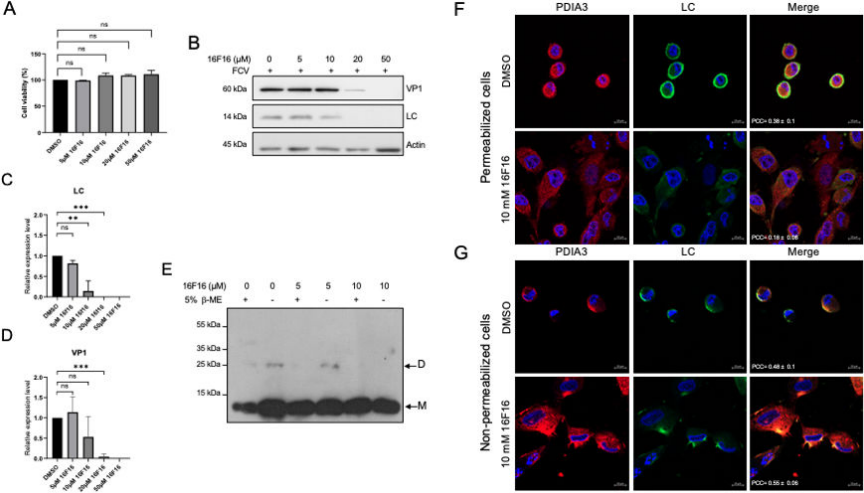
PDI inhibitors reduce LC protein levels and oligomer formation and modify LC protein subcellular localization. (A) CrFK cells were treated with 5, 10, 20, and 50 µM of 16F16, and cell viability was assessed by an MTT assay. (B) Total protein extracts from mock-infected or FCV-infected cells at an MOI of 5 for 5 h, untreated or treated with 5, 10, 20, and 50 µM of 16F16, were obtained, and levels of LC and VP1 proteins were determined by western blotting. Actin was used as a loading control. (C) LC and (D) VP1 band intensities from scanned images were quantified using ImageJ software and shown as relative expression. Standard deviations were obtained from three independent experiments. Values of *P* < 0.000 (***) calculated by *t*-test using GraphPad Prism 8.0 software are indicated. (E) Total protein extracts from FCV-infected cells at an MOI of 5 for 5 h, untreated (0) or treated with 5 and 10 µM of 16F16, were obtained and analyzed by SDS-PAGE in the presence (+) or absence (−) of 5% β-ME. The presence of the LC protein monomers (M) and dimers (D) was determined by western blotting. (F) Permeabilized and (G) non-permeabilized mock-infected and FCV-infected cells at an MOI of 5 for 5 h and treated with DMSO or 10 µM of 16F16 were immunostained with an anti-LC serum (green) and anti-PDIA3 (red) antibodies. DAPI was used for nuclear (blue) staining. The cells were examined in a Zeiss LSM 700 confocal microscope. Images correspond to a z-stack of 15 slices and represent at least three independent experiments. Merged images are indicated. Pearson’s correlation coefficients, indicated in the images, were calculated for 25 individual cells from two independent experiments. Data are presented as mean ± SD using Zeiss software (https://www.zeiss.com.mx/corporate/home.html).

To determine if PDIs have a role during the FCV replicative cycle, their function was inhibited with the 16F16 compound (38). First, the viability of CrFK cells treated with 5, 10, 20, and 50 µM concentrations of 16F16 for 5 h was evaluated by MTT colorimetric assays (Fig. 8A). Since no changes in cell viability occurred in the presence of the 16F16 at the tested concentrations, the effect of PDI inhibition in viral protein production was analyzed. Total protein extracts from 5, 10, 20, and 50 µM of 16F16-treated FCV-infected cells were obtained, and both VP1 and LC protein levels were analyzed by western blotting (Fig. 8B through D). A statistically significant reduction in the LC protein levels from 10 to 50 µM was observed when compared to the DMSO control (Fig. 8C); moreover, a statistically significant reduction in the VP1 protein levels was observed at 20 and 50 µM of 16F16 (Fig. 8D), suggesting that PDI functions are important for FCV replication.

To further analyze if PDI function is involved in the LC protein homodimer formation and in its subcellular localization, 16F16-treated cells were infected with FCV and analyzed by western blotting (Fig. 8E) and immunofluorescence assays (Fig. 8F and G). The role of PDIs in the formation of LC protein oligomers was determined in infected cells treated with increasing concentrations of the PDI inhibitor 16F16. While the presence of the monomer (M) was observed under all conditions tested (Fig. 8E), dimer (D) formation was detected only at 0 and 5 µM 16F16. The lack of dimer formation at 10 µM 16F16 indicates that it requires the activity of a PDI. The 28 kDa band corresponding to the LC protein dimer was not observed in the presence of 5% β-ME, corroborating that LC oligomerization occurs through disulfide bonds. On the other hand, to determine if PDIs were involved in the LC protein subcellular localization, immunofluorescence assays were performed in the presence of 10 µM 16F16. While DMSO-treated FCV-infected cells for 5 h showed the typical cytopathic effect (Fig. 8F), no cell rounding was observed in 10 µM 16F16-treated infected cells (Fig. 8F). The LC protein signal is less intense in 16F16-treated cells in comparison to the DMSO-treated cells (Fig. 8F), in concordance with the reduction in LC protein levels observed in western blotting assays (Fig. 8B). Moreover, colocalization between the LC protein and PDIA3 was significantly decreased in the presence of 10 µM 16F16 in permeabilized cells, with a Pearson’s coefficient colocalization value of 0.18 ± 0.08 (Fig. 8F and 7A). To determine if the LC protein was able to reach the outer face of the plasma membrane when PDIs were inhibited, the LC protein localization was determined in non-permeabilized cells (Fig. 8G). Again, we found that the cytopathic effect in 16F16-treated infected cells was not as evident as in the DMSO-treated infected cells; however, the LC protein localization remained focalized in one side of the outer face of the plasma membrane and associated with PDIA3 in both 16F16 and DMSO-treated cells, with a Pearson’s coefficient colocalization value of 0.55 ± 0.08, (Fig. 8G), suggesting that colocalization between PDIA3 and the LC protein is not affected by PDI inactivation (Fig. 8G and 7B). These results, taken together, suggest that PDIA3 plays a role in the LC protein stability and intracellular distribution during FCV replication and in the LC protein dimer formation, but not in its translocation to the outer face of the plasma membrane from the infected cells.

## DISCUSSION

The LC protein is unique to the members of the genus *Vesivirus* in the family *Caliciviridae* and is essential for successful viral replication. It is encoded in the open reading frame (ORF) 2 and expressed from the sgRNA as the LC-VP1 precursor protein that is further processed by the viral protease-polymerase NS6/7, and it has not been found to be present in the mature viral particles. Although the LC protein from FCV was identified over 30 years ago, its importance in FCV infection was established by Abente et al. in 2013, who found that the whole protein is needed for successful viral replication. They identified two conserved regions (CRs) necessary for its function and its interaction with the cellular factor annexin A2, which our workgroup further identified as being implicated in efficient FCV replication (14). Even though it was identified that the LC protein participates in establishing the cytopathic effect, its function and mechanism of action still need to be fully elucidated.

The first clue suggesting that the LC protein from FCV acts as a viroporin came from our research group. Barrera et al. determined that the LC protein expression in a virus-free context resulted in its localization to the mitochondria, which induced periplasmic protein relocalization to the cytosol and triggered apoptosis, similar to other viroporins from RNA viruses (39). Furthermore, our research group reported that the purified LC recombinant protein forms homo-oligomers through disulfide bonds, and its expression is toxic in *Escherichia coli* cells, likely through osmotic stress marked by plasmolytic bay formation, suggesting that the LC protein from FCV is a viroporin (1). We further hypothesized that disulfide bond formation in the LC protein may play a role in its toxicity and permeation capabilities, like the Ebola virus (EboV) delta peptide viroporin (34). More recently, we reported that the purified recombinant LC protein can permeate the plasma membrane when interacting exogenously to non-infected cells, which is another characteristic of viroporins (17). However, the concentration and incubation period of LC protein required to permeate the cytoplasmic membrane of CrFK cells (20 and 30 µM for at least 5 h) are considerably higher than levels of extracellular LC protein during infectionin the *in vitro* system used to detect its secretion. Further studies are needed to confirm the secretion of LC from FCV-infected cells under physiological conditions and to evaluate its potential to permeate neighboring cells and contribute to viral pathogenesis.

Viroporins are virus-encoded transmembrane proteins that form pores or ionic channels and participate in multiple steps of viral replication in a virus-specific manner, making them attractive drug targets to minimize virus pathogenesis and replication (40). Our previous works demonstrated that the LC protein from FCV has common viroporin characteristics (16). In this work, we aimed to characterize the LC viroporin during FCV infection in cell culture. To this end, immunized mice serum was obtained to determine LC protein expression and subcellular localization throughout the FCV replication cycle. The LC protein expression from FCV was first detected by western blotting from 3 and up to 9 hpi, with the highest levels achieved at 5 hpi, similar to the VP1 expression kinetics. When analyzing the subcellular localization of the LC protein in infected cells by immunofluorescence, we did not find it in the mitochondria, as when expressed in a virus-free system, which suggests that the LC protein may not play a direct role in the apoptosis induction through the intrinsic pathway, as reported in FCV infection (41–43). However, we cannot rule out an indirect role in apoptosis induction during infection. Here, we found that a predominant localization of the LC protein during infection was observed in the inner periphery of the infected cells, suggesting that this differential subcellular localization requires the presence of other viral proteins, needs a post-translational modification, or is the result of the infected cell context.

The localization of the LC protein from FCV on both the inner and probably the outer face of the plasma membrane suggests that it could be involved in modulating cell surface signaling pathways. As previously discussed by Abente et al. (13), the CRII of the LC protein consists of conserved polyproline residues, which are common SH3 domain-binding motifs. Identifying potential interactions with SH3 domain-containing proteins may help to elucidate a possible role for the LC protein in modulating host-cell signaling pathways during infection. The moderate colocalization between LC and the cytoskeletal protein actin at the inner face of the plasma membrane, observed in some FCV-infected cells, may also suggest a potential spatial association. Although not strongly colocalized, this observation is consistent with a possible role for the LC protein in cytoskeletal rearrangement, in accordance with its previously reported role in the establishment of the cytopathic effect through its CR domain. Whether the cytopathic effect is a consequence of the LC protein interaction with cytoskeletal proteins remains to be determined.

A way in which proteins can be associated with the cell membrane is through lipidation as a post-translational modification. By *in silico* analysis, we predicted that LC contains (i) putative palmitoylation sites located at amino acids 2, 5, 39, and 40 (N-terminal end), with residues 2 and 5 as the most likely candidates (Fig. 5A), and (ii) a TMD located between amino acids 98 and 113 (C-terminal end) (17). Thus, it was possible that palmitoylation of the LC protein enables the TMD to associate more tightly with the plasma membrane, as has been described for HIV and simian immunodeficiency virus (SIV) envelope glycoproteins (44). Although there were no previous reports of viral proteins in the family *Caliciviridae* undergoing this modification, our *in silico* prediction was the first hint suggesting that the FCV LC viroporin could be palmitoylated during infection. This was further demonstrated with the ABE method (Fig. 5), with LC being the first caliciviral protein reported to be palmitoylated. The effect of the palmitoylation inhibition with 2-BP in FCV-infected cells resulted in an alteration of the LC protein subcellular localization from the inner side of the cell membrane to a more homogeneous cytoplasmic distribution and in a reduction of the LC protein levels, which could also be the cause of the change in the LC protein distribution. This result resembles the changes that palmitoylated viral proteins undergo when this lipidation is inhibited, such as the TF protein of the Sindbis virus (45) and the NS2 protein of the hepatitis C virus, and even in other viroporins like ORF3 of hepatitis E virus (46) and other viral proteins (reviewed in reference 29), suggesting that palmitoylation is involved in the LC protein localization to the inner face of the cell membrane or in its proper folding and interactions with other cellular or viral proteins, as has been suggested for the HIV and SIV envelope glycoproteins (44).

Interestingly, Abente et al., in 2013, demonstrated that insertions such as mKate fluorescent protein at position 88 of the LC protein are tolerated and do not impair LC expression or its cytopathic activity in cells (13). Since the mKate insertion site is located downstream of the putative palmitoylation sites and upstream of the TMD, it is unlikely to interfere with the palmitoylation process itself. However, this insertion may influence the proposed tighter association with the plasma membrane or alter the subcellular localization of LC, although this possibility remains to be experimentally validated in future studies.

On the other hand, we have found that the possible localization of the LC protein to the external face of the membrane of FCV-infected cells in the absence or presence of 2-BP remained unchanged, even in the treated infected cells that did not exhibit the typical cytopathic effect. This suggests that palmitoylation is primarily involved in the intracellular localization of the LC protein. The palmitoyl-transferase enzyme(s) responsible for this lipidation, as well as the importance of this post-translational modification in the LC protein function inside the infected cell, remains to be determined.

As the LC protein seems to be focalized in the outer face of the cytoplasmic membrane of FCV-infected cells, we hypothesized that this protein was secreted during viral replication, similar to viroporins from other virus families like NSP4 from human rotavirus (47) and delta peptide from EboV (28). The presence of the LC protein in the extracellular environment prior to the onset of apoptosis indicates that it is secreted and suggests that its function during the FCV replication cycle is as an extracellular viroporin. This is the first report of a secreted calicivirus protein that has viroporin functional characteristics. Although the secretion of extracellular vesicles containing virions and viral RNA from FCV-infected cells has been previously reported (48), the role of free or vesicle-associated secreted FCV proteins has not been thoroughly studied, representing an attractive research line to better understand the establishment of the FCV pathogenesis.

As the LC protein from FCV forms disulfide bond-dependent homo-oligomers in FCV-infected cells, we hypothesized that disulfide isomerase proteins might mediate this process. However, LC was not detected in the ER or the Golgi apparatus, the main subcellular compartments in which these catalytic proteins normally reside. PDIA3, a disulfide isomerase localized predominantly in the ER, is also present in other cellular compartments, including the cytoplasm and nucleus, where it contributes to protein folding, redox regulation, signaling, and additional cellular processes (19). Moreover, PDIA3 is involved in the replication of several viruses (18–22). The discrete colocalization observed between the LC protein and PDIA3 at 5 hpi on both sides of the plasma membrane, along with the confirmation of their interaction by co-immunoprecipitation assays (Table 1 and Fig. 7C), strongly suggests that the LC protein from FCV interacts with this cellular chaperone to facilitate its proper folding and/or oligomerization. Inhibition of PDIs using 16F16 resulted in a reduction of the VP1 and LC protein levels and induced an alteration in the LC protein intracellular localization in FCV-infected cells, but not in its focalized localization at the plasma membrane. Moreover, the LC protein dimers’ formation depends on disulfide bonds, probably through PDI’s function. Although disulfide bond formation is generally unfavorable in the reducing environment of the cytoplasm (49), oxidative stress, such as that induced during FCV infection, can impair the glutathione protein reducing system and enable thiol bond formation. Apoptosis, widely known to be triggered by FCV (41, 42), alters the cellular redox state and may thus promote LC protein dimerization via disulfide bonds (50), as observed for some proteins including the rotavirus μ protein (51). Further studies are needed to understand the dynamics and functional relevance of this process during FCV replication cycle. The fact that the levels of both VP1 and LC proteins were reduced when PDI function was inhibited by 16F16, with no effect on cell viability, suggests that this compound (or other PDI inhibitors) could be used as an antiviral drug, as previously explored for other viruses (52). The downregulation of PDIA3 results in apoptosis triggering in several cell lines and is reported to be overexpressed in several cancer types, making it a potential biomarker (53) and drug target (54, 55). Therefore, PDI inhibition by 16F16 could also impact virus production through apoptosis modulation.

**Fig 9:**
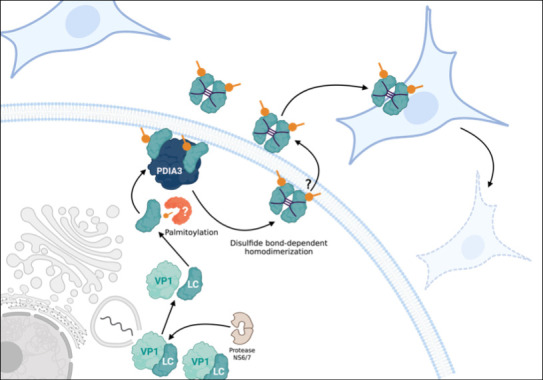
Proposed model for LC protein palmitoylation, dimerization, and secretion during FCV infection. Once the VP1-LC precursor is produced and proteolytically processed by the protease-polymerase NS6/7 during the late stages of the FCV infection, some mature LC proteins become palmitoylated and reach the plasma membrane, where they interact with PDIA3 to promote LC homodimerization through disulfide bonds. These LC homodimers are then secreted and interact with neighboring cells, permeabilizing their membranes.

These results, taken together, elucidated the expression, kinetics, and subcellular localization of the LC protein, as well as novel characteristics during the FCV replication cycle, such as its palmitoylation and association with PDIA3, a disulfide isomerase involved in the replication of several viruses (18–22), which may be related to the LC protein dimer formation, as is the case for the oligomerization of the hemagglutinin protein of influenza virus (IAV) (20). Moreover, the fact that the LC protein is secreted during infection might be another viroporin characteristic, as has been reported for rotavirus NSP4 (56) (Fig. 9). The characterization of the LC protein in FCV-infected cells will provide new research lines to better understand this unique protein and its role during infection and will help develop strategies to control and prevent FCV infection.

## MATERIALS AND METHODS

### Cell culture and viral infection

CrFK cells were obtained from the American Type Culture Collection (Rockville, MD) and cultured in minimal essential medium (MEM) Advance supplemented with 5% fetal bovine serum (FBS) (Gibco), 5,000 U of penicillin, and 5 µg/mL of streptomycin. Cells were incubated at 37°C in 5% CO_2_. FCV Urbana strain was produced from the reverse genetic system using the pQ14 infectious clone kindly provided by Dr. Green (Laboratory of Infectious Diseases, NIAID, NIH, Bethesda, MD) (57).

Virus titers were quantified by plaque assay as described (58). The virus was diluted in MEM and used to infect 90% confluent CrFK cells at an MOI of 5, following two phosphate-buffered saline (PBS) washes (137 mM NaCl, 2.7 mM KCl, 10 mM Na_2_HPO_4_, and 1.8 mM KH_2_PO_4_) at 37°C for 5 min. Viral adsorption occurred over a 1 h incubation at 37°C in 5% CO_2_, with gentle rocking every 15 min. After removing the viral inoculum, cells were washed with PBS at 37°C and incubated as previously described.

### Immunofluorescence assays

CrFK cells were grown on coverslips and infected as previously described. After infection and/or pharmacological treatment, cells were fixed with 3.7% paraformaldehyde (vol/vol) in PBS for 20 min and permeabilized with 0.1% Triton X-100 in PBS (vol/vol) for 5 min (permeabilization was omitted for non-permeabilized cells). Blocking was performed with 0.5% porcine skin gelatin for 30 min, followed by overnight (ON) incubation with the corresponding primary antibodies in PBS. Secondary Alexa 488 and CY5 fluorophores (Thermo Fisher Scientific) were diluted 1:200 in PBS and incubated for 2 h at room temperature. Cells were stained with DAPI (4′,6-diamidino-2-phenylindole dihydrochloride) (Thermo Fisher Scientific) in PBS for 5 min. Mounting was done with VectaShield (Vector Laboratories), and analysis was performed using a Zeiss LSM 900 confocal microscope with ZEN lite software. All images were taken as optical sections in the *z*-axis unless otherwise noted.

The antibodies for confocal microscopy and their respective dilutions were the following: anti-LC (mouse serum, lab-generated) 1:120; 1:80; anti-annexin A2 (lab generated) 1:100; anti-PDIA3 (ABClonal Technology) 1:110; anti-PDI (AbClonal Technology) 1:100; anti-GM130 (GeneTex) 1:130. MitoTracker Deep Red (Invitrogen) and rhodamine-phalloidin (Thermo Fisher Scientific) were used for actin staining according to the manufacturer’s instructions.

### Plasmid purification and transfection

CrFK cells grown on coverslips in six-well plates (Corning) to 70% confluence were transfected with 3.5 µg of pAm-Cyan and Wt-LC-pAm-Cyan plasmids (15), using Lipofectamine 2000 (Thermo Fisher) in MEM medium (Gibco) following the standard transfection protocol. The transfected cells were then processed for immunofluorescence assays after 48 h.

### Recombinant protein expression

Chemo-competent *E. coli* BL21(DE3)pLysS, transformed with pRSETA-LC plasmid (16), were grown in Luria-Bertani broth (LB) medium (NaCl 0.17M, 1% peptone, 0.5% yeast extract) with ampicillin (100 mg/µL) at 37°C and 200 rpm until OD_600_ reached 0.4. Induction was done with isopropyl β-D-1-thiogalactopyranoside (Thermo Fisher Scientific) at a final concentration of 0.1 mM. Six hours post-induction, cells were sonicated following QIAexpressionist (Qiagen) protocols 9 and 10 using 8 M urea for protein denaturation. Membrane fractions were buffer-exchanged to PBS with Amicon Ultra 2 mL filters (Merck Millipore) and purified via fast protein liquid chromatography (FPLC) with a Ni^2+^ column (Thermo Fisher).

### Animal immunization and serum purification

BL6 mice were immunized with 50 µg of the His-tagged LC purified protein in PBS and TiterMax (Sigma-Aldrich) adjuvant (1:10 ratio) over four immunizations, each 14 days apart. Mice were euthanized via cardiac puncture, and serum was purified by differential centrifugation.

### Total protein extracts from CrFK cells

CrFK cells treated under specified conditions (infection, drug treatment or both) were harvested using plastic scrapers, pelleted at 1,500 relative centrifugal force (RCF) for 5 min at 4°C, washed with pre-cooled PBS, and resuspended in RIPA buffer (150 mM NaCl, 1% Nonidet N-P40, 0.5% deoxycholate, 0.1% SDS, 50 mM Tris-HCl, pH 7.4). The lysates were incubated for 60 min at room temperature (RT), centrifuged at 20,000 RCF for 30 min at 4°C, and the supernatants were collected. Protein degradation was minimized using cOmplete protease inhibitor cocktail (Roche), 5 mM EDTA, and 0.1 mM phenylmethylsulfonyl fluoride (PMSF). Protein concentration was determined using a BCA Protein Asaay Kit (Thermo Scientific), and 10 µg of protein was used per lane for western blotting. For non-reducing SDS-PAGE, Laemmli buffer lacked β-ME.

### SDS-PAGE and western blotting

SDS-PAGE followed the Bio-Rad Bulletin no. 6040 guide. Proteins were transferred to a nitrocellulose membrane (Bio-Rad) using Dunn buffer (10 mM NaCHO_3_, 3 mM Na_2_CO_3_, 8% methanol). Membranes were stained with Ponceau red, blocked with PBS-0.1% Tween 20 and 5% skimmed milk for 30 min at RT, and incubated with primary antibodies in PBS-Triton X-100 (0.1%) (Sigma) ON at 4°C. Secondary antibodies conjugated with HRP (Jackson Immunoresearch) were diluted 1:10,000 in PBS-Tween or PBS-Triton X-100 with 1% skimmed milk for 2 h at RT. Membranes were washed twice with PBS-Tween or Triton X-100 and developed using Super Signal West Femto substrate (Thermo Scientific) and Carestream X-ray films.

#### Primary antibodies used

Α-actin (Santa Cruz Biotechnology): 1:80,000. Anti-Vp1 (mouse serum, lab-generated): 1:100,000 (37). Anti-LC (mouse serum, lab-generated): 1:60,000. Anti-PDIA3 (ABClonal Technology): 1:10,000.

### *In situ* cell fixation for western blot analysis

CrFK cells were infected and harvested by scrapping, centrifuged at 300 RCF for 5 min at 4°C, resuspended in ice-cold PBS, and centrifuged again. The supernatant was discarded, and cells were resuspended in PBS with paraformaldehyde (PFA) at the indicated concentrations, incubated with agitation for 20 min at RT, centrifuged, and resuspended in RIPA buffer for lysis and western blotting analysis.

### Pharmacological treatments

2-BP was dissolved in DMSO at specified concentrations. Post-infection, cells were washed with PBS and incubated in MEM with 2% FBS and either 2-BP or 1% DMSO. For 16F16 (Santa Cruz Biotechnology), resuspended in DMSO to 0.311 M and stored at −80°C, cells were treated similarly.

### Prediction of palmitoylation sites

S-palmitoylation sites on the FCV LC protein were predicted using GPS-Palm 4.0 (https://gpspalm.biocuckoo.cn) in November 2022.

### ABE assay

Detection of S-palmitoylation of the LC protein from FCV by ABE assay was performed as previously described (32). Briefly, the cells were lysed in the presence of 50 mM N-ethylmaleimide (Sigma) with lysis buffer containing 1% IGEPAL (Sigma), 50 mM Tris-HCl, pH 7.5, 150 mM NaCl (Thermo Fisher), and 10% glycerol (Thermo Fisher). cOmplete EDTA-free protease inhibitor cocktail (Roche) and 0.1 mM PMSF (Sigma) were used as protease inhibitors. The target protein was immunoprecipitated with anti-LC serum for 2 h at RT, and agarose beads coupled with protein A+G (Santa Cruz) were incubated at 4°C, ON under rocking conditions. The immunocomplex was incubated with 1 M HAM (Sigma) in lysis buffer at pH 7.2 for 1 h at RT and washed three times. Biotinylation of the target protein was performed with Biotin-HPDP (Santa Cruz) in lysis buffer at pH 7.2 at 4°C for 1 h and washed three times. The samples were heated at 80°C for 10 min and centrifuged at 13,000 × *g* for 3 min to pellet the agarose beads. The samples were analyzed with SDS-PAGE and detected using streptavidin-HRP (Thermo Fisher) or anti-LC serum as previously described.

### Membrane permeation assessment through Sytox Green entry assay

CrFK cells were seeded in 96-well culture plates until 80%–90% confluence and mock-infected or infected with FCV at an MOI of 5. At the indicated times, 50 nM of Sytox Green diluted in a culture medium without SFB was added and incubated for 10 min before fluorescence measurement. The fluorescence intensity was determined at 1, 3, 5, 7, and 9 hpi with the fluorometer Fluoroskan Ascent FL (Thermo Fisher). Each measure was compared with the values obtained at 0 hpi.

### Protein precipitation from the cell supernatant

Protein precipitation was performed as previously described (59). Briefly, supernatants of CrFK cells, mock-infected or infected with FCV at a MOI of 5, were collected, and cell debris was removed by centrifugation at 500 RCF at 4°C for 5 min, discarding the pellet, and at 2,000 RCF at 4°C for 30 min, discarding the pellet. Finally, centrifugation at 20,000 RCF at 4°C for 30 min was performed, keeping the supernatants. Five hundred microliters of methanol (J.T. Baker) and 125 µL of chloroform (J.T. Baker) were added to 500 µL of supernatants and mixed by vortexing until a homogeneous solution was formed. The samples were centrifuged at 13,000 RCF for 5 min. The aqueous phase was carefully removed using a vacuum pump, and 500 µL of methanol was added before mixing by vortexing until the protein pellet was broken down. The samples were centrifuged at 13,000 RCF for 5 min, the aqueous phase was discarded using a vacuum pump, and the pellet was resuspended in RIPA lysis buffer (10 mM Tris-Cl, 1 mM EDTA, 0.5 mM EGTA, 1% Triton X-100, 0.1% sodium deoxycholate, 0.1% SDS, 140 mM NaCl at pH 8) with cOmplete EDTA-free protease inhibitor cocktail (Roche) for SDS-PAGE and western blotting analysis.

### LC co-immunoprecipitation assays and tandem mass spectrometry (MS/MS) analysis

A total of 7 × 10^7^ CrFK cells, non-infected or infected with FCV at an MOI of 5 for 5 h, were harvested with a scraper and washed once with ice-cold PBS before RIPA lysis and protein extraction, as previously described. Total protein extracts were incubated with agitation with 2.3 µL α-LC mouse serum for 2 h at RT. Nine microliters of protein A/G plus agarose beads (Santa Cruz Biotechnology) was added and incubated at 4°C ON. Samples were centrifuged at 500 RCF at 4°C for 5 min and washed two times with PBS. Protein was eluted in Laemmli buffer 2× at 95°C for 5 min for SDS-PAGE and western blotting analysis. For protein mass spectrometry, immunoprecipitated samples from mock-infected and infected cells were resolved in a 10% SDS-PAGE at 80 V and then allowed to advance for about 1 cm within the gel. The polyacrylamide gel was stained with Coomassie Blue (Bio-Rad), and the resulting gel fragments were enzymatically digested and analyzed by MALDI-TOF/TOF (MS/MS) at the “Unidad de Genómica, Proteómica y Metabolómica, at Cinvestav, Mexico”. Generated *.raw files were deconvoluted and compared using ProteinLynx Global SERVER v.3.0.3 (Waters, Milford, MA) against a reversed *Felis silvestris catus* UP000011712 *.fasta database (downloaded from UniProt, 40,213 protein sequences, revised in March 2023) and *Feline calicivirus* UP000001098 *.fasta database (downloaded from UniProt, three protein sequences, revised in March 2023) concatenated with its reverse database.

### MMT assay

CrFK cells were plated in 96-well culture plates (10,000 cells per well) in Dulbecco’s modified Eagle’s medium supplemented with 5% FBS until 90% confluence. Drug treatment was given according to the experimental requirement. The medium was removed, and 100 µL of fresh medium was added with 10 MTT (Sigma) to a final concentration of 1.2 mM; the cells were incubated for 4 h at 37°C and protected from light. The formazan crystals formed by the cells were dissolved by adding 100 µL of 5% SDS (Bio-Rad) in 1N HCl (Thermo Fisher) and incubated for 12 h at 37°C. Finally, the absorbance was measured at 540 nm using a microplate reader.

## Data Availability

All data generated or analyzed during this study, including Western blot images, immunofluorescence micrographs, plaque assay results, and schematic figures created with BioRender, are available from the corresponding author upon reasonable request. No data sets were deposited in public repositories.
